# Synergistic Enhancement of Compromised Skin Radiance: A Clinical Investigation of *Prinsepia utilis Royle* Polysaccharides and Nonapeptide Co‐Application

**DOI:** 10.1111/jocd.70204

**Published:** 2025-05-14

**Authors:** Chong Qin, Liu Zhang, Ruiqi Zhang, Bo Wang, Qin Zhang, Yannan Bian, Wenying Liu, Li He, Feifei Wang

**Affiliations:** ^1^ Yunnan Characteristic Plant Extraction Laboratory Yunnan Yunke Characteristic Plant Extraction Laboratory Co., Ltd. Kunming Yunnan China; ^2^ Shanghai Jiyan Biomedical Development Co., Ltd. Shanghai China; ^3^ Yunnan Botanee Bio‐Technology Group Co., Ltd. Kunming Yunnan China

**Keywords:** ESETRILLQ peptide, genetic analysis, placebo‐control, *Prinsepia utilis Royle* polysaccharides, skin barrier, skin radiance

## Abstract

**Background:**

Skin radiance represents both healthy and esthetic aspects of human skin, usually influenced by a compromised barrier and the aging process. The reduction of the stratum corneum by chemical peels is a prevalent procedure employed to enhance facial radiance, but peeling is not suitable for compromised skin.

**Objectives:**

*Prinsepia utilis Royle* polysaccharides (PURP) is a natural extract with repairing properties, which has been reported as a barrier repairing agent. ESETRILLQ (EQ) peptide has been recently reported as a novel antiaging bioactive peptide. This study aims to investigate the combined efficacy of these two ingredients on skin radiance enhancement.

**Methods:**

Reconstructed human full‐thickness skin models were subjected to UVA exposure, followed by treatment with 1000 ppm PURP, 20 ppm EQ9, and their combinations: PUR9‐1 (1000 ppm PURP + 10 ppm EQ9) and PUR9‐2 (1000 ppm PURP + 20 ppm EQ9). Transcriptomic profiling was performed as a preliminary study to define the synergistic effect. RT‐qPCR was performed assessing the regulation of skin barrier‐related genes. Thirty‐three Chinese sensitive skin individuals were enrolled in a placebo‐controlled split‐face clinical research for 2 weeks to evaluate a PUR9‐2 containing lotion. Instrument measurement and expert evaluation were conducted to evaluate the parameters of glossiness and skin tone at baseline, Day 7, and Day 14. Skin glossiness was determined by VISIA 7, Glossymeter, and Translucency Meter. TEWL was determined by Tewameter Hex. Wrinkel number and area were obtained by VISIA 7.

**Results:**

Transcriptomic profiling identified PUR9‐2 to regulate significantly different genes distinct from PURP and EQ9. The combination increased the gene expression levels of TNFAIP3 and CRNN. PUR9‐2 also increased the expression of FLG, LOR, and DSG1on UVA‐irradiated skin model. PUR9‐2 containing lotion significantly decreased TEWL by 16.96%. Clinical evaluations demonstrated a statistically significant 22.32% (Glossymeter) and 35.56% (VISIA 7) improvement in skin glossiness on the PUR9‐2 lotion‐treated side by Day 14 compared to baseline. Translucency demonstrated a statistically significant 13.06% increase of *K* value, which all aligned with the expert evaluation of skin radiance enhancement.

**Conclusion:**

PCA analysis revealed PUR9‐2 uniquely modulated gene expression compared to PURP and EQ9. Functional enrichment analysis based on Gene Ontology (GO) demonstrated PUR9‐2 restored UVA‐suppressed TNFAIP3 and CRNN gene. The results of RT–PCR also indicated that PUR9‐2 enhanced skin barrier integrity in 3D models via upregulated expression of FLG, LOR, and DSG1. The Chou‐Talalay method further validated PUR9‐2's synergistic potency (CI < 1) in accelerating keratinocyte scratch wound closure. The clinical research demonstrated protective effects of PUR9‐2 on compromised skin barrier and enhanced both glossiness of the sensitive skin surface and translucency within the skin structure. This study provides a potential solution for improving the radiance and overall conditions of compromised skin.

## 1 Introduction

Skin radiance is closely affected by factors such as skin barrier disturbance and aging [1, 2]. In practice, the reduction of the stratum corneum by chemical peels is a prevalent procedure employed to enhance facial radiance [3]. However, peeling is not suitable for compromised skin [4]. In the realm of cosmeceutics, *Prinsepia utilis Royle* polysaccharides (PURP) are a natural botanical extract derived from *Prinsepia utilis Royle*, a plant endemic to southwestern China. PURP have been shown to reinforce the epidermal integrity by enhancing claudin protein expression in 3D skin models at concentrations of 2000–5000 ppm [5]. The novel nonapeptide ESETRILLQ (EQ9), identified via virtual screening of the regenerative cell secretome, demonstrates potential antiaging effects by modulating collagen I, MMP‐9, and ROS in fibroblast cell models at concentrations as low as 10–100 ppm [6]. This study aims to explore a gentle approach to enhance facial radiance in populations with compromised barriers by combining PURP and EQ9.

Transcriptomic profiling is a technology in modern dermatological research that provides a comprehensive view of the molecular mechanisms involved in the skin's response to disease and environmental factors (ex. UV irradiation) [7]. Epidermal keratinocytes have been extensively studied using transcriptomic profiling to identify the changes occurring during epidermal differentiation and cornification. Reconstructed full‐thickness skin models, when combined with transcriptomic profiling, allow us to study the behavior of skin cells in a controlled environment (such as UV radiation) that mimics an UV‐induced compromised skin [8].

Individuals with sensitive skin often exhibit inflammatory responses and compromised skin barriers, which are provoked by exaggerated neurovascular reactions and immune system responses to both internal and external stimuli [9]. The lactic acid sting test (LAST), which assesses skin barrier integrity by inducing pruritus and stinging sensations via activation of TRPV1 and CGRP receptors, is commonly used to screen for sensitive skin [10, 11, 12].

In this study, we identified a combination PUR9‐2 (1000 ppm PURP and 20 ppm EQ9) by transcriptomic profiling to unravel its potential efficacy, followed by in vitro validation of PUR9‐2's effect on compromised skin. We then investigated the effects of PUR9‐2 on improving the sensitive skin radiance by conducting a clinical study on 33 individuals with sensitive skin selected by the lactic acid sting test. This study aimed to assess the efficacy of PUR9‐2 in improving skin radiance in compromised skin, providing a gentle therapeutic solution to address skin dullness associated with barrier impairment.

## 2 Materials and Methods

### 2.1 3D Full Thickness Skin Model and Treatment

T‐Skin is an in vitro reconstructed skin which consists of a dermal equivalent with human fibroblasts overlaid by a stratified, well‐differentiated epidermis derived from normal human keratinocytes cultured on an inert polycarbonate filter (Shanghai Si an Funuo Biotechnology Co. Ltd., Shanghai, China). T‐skin models were stably cultured in T‐skin medium for 24 h prior to UVA irradiation and treatment with ingredients. T‐skin models were irradiated with UVA at a daily dose of 2 J/cm^2^ for two consecutive days (total irradiation dose: 4 J). After each irradiation session, models were treated with 1000 ppm PURP, 20 ppm EQ9, combination PUR9‐1 (1000 ppm PURP +10 ppm EQ9) and combination PUR9‐2 (1000 ppm PURP +20 ppm EQ9). All models were subsequently placed in a CO_2_ incubator (37°C, 5%CO_2_) for incubation. After incubation, the tissues were washed by PBS. Based on previous studies, tissue viability of skin models treated with 1000 ppm (1 mg/mL) PURP was over 80%, and 100 ppm EQ9 was not cytotoxic to HaCat cells [5, 6].

### 2.2 RNA Extraction and Transcriptomic Analysis

Total RNA was extracted from the T‐skin models (including epidermis and dermal equivalent), using Trizol reagent (Invitrogen) following the manufacturer's procedure. High‐quality RNA samples with RIN number > 7.0 were used to construct sequencing libraries on the Illumina Novaseq 6000, generating paired‐end sequencing of 150 bp in length. After the final transcriptome was generated, StringTie and ballgown (http://www.bioconductor.org/packages/release/bioc/html/ballgown.html) were used to estimate the expression levels and perform expression abundance for mRNAs by calculating fragment per kilobase of transcript per million mapped reads (FPKM) value. Differential gene expression analysis was performed using DESeq2 by https://www.bioinformatics.com.cn and visualized by http://www.sangerbox.com/tool. For functional enrichment analysis, we then utilized the biological processes (BP) of gene ontology (GO) [13] enrichment and implemented the analysis using Metascape online tools.

### 2.3 Quantitative RT–PCR

Total RNA was extracted using Trizol reagent (Invitrogen) following the manufacturer's procedure. First‐strand complementary DNA (cDNA) was synthesized. A Roche LC96 was used to perform quantitative RT–PCR with SYBR Green I master mix. The QRT‐PCR results are shown as the relative expression levels normalized to the expression of β‐actin, which was used as an internal reference (β‐actin‐F/R), and three biological replicates were included for each experiment.

### 2.4 Melanin Inhibition Assay

B16F10 cells are murine melanoma cells that synthesize and secrete melanin under normal physiological conditions. Phenylthiourea (PTU) was used to inhibit melanogenesis as a positive control. The method for melanin synthesis inhibition was described by Nouha Nasr Bouzaiene [14]. The effects of PUR9‐1 and PUR9‐2 on melanogenesis are listed in Figure S1.

### 2.5 Antioxidation Assay

The antioxidant testing adopted the method invented by Nishikimi, which utilizes the xanthine–xanthine oxidase system to generate superoxide anion radicals. This method tests the inhibitory effect of the substance on superoxide anion radicals and employs the proven excellent superoxide anion scavenger vitamin C (150 ppm) as a positive control [15]. The antioxidant effects of PURP, EQ9, and PUR9‐2 are listed in Figure S2.

### 2.6 In Vitro Scratch Wound Healing Assay

The method for scratch wound healing assay was described by Simona Martinotti [16]. The HaCaT cell line was obtained from the National Infrastructure of Cell Line Resource, Shanghai, China. The repairing effects of PURP (1000, 500, and 250 ppm), EQ9 (20, 10, and 5 ppm), and their combo (1000 ppm PURP +20 ppm EQ9; 500 ppm PURP +10 ppm EQ9; 250 ppm PURP +5 ppm EQ9) were evaluated. The combo was formulated with PURP and EQ9 at a constant ratio of 50:1 (w/w). Percentages of cell‐covered area were quantified at 48 h to obtain dose–effect curves and combination index (CI) values. CI values were calculated using CompuSyn software (Chou‐Talalay method) [16].

### 2.7 Placebo‐Controlled Split‐Face Clinical Research

#### 2.7.1 Participants Inclusion

The efficacy of PUR9‐2 was investigated in the model of UVA‐induced T‐Skin skin with a compromised barrier and increased inflammation. This model was then used to investigate the synergistic effects of PUR9‐2 in skin barrier repair. Therefore, sensitive skin, characterized by barrier damage and inflammation [1, 2, 17, 18, 19], was selected to investigate the efficacy of PUR9‐2 in clinical studies. Sensitive skin was identified through a lactate stinging test. A cohort of 33 healthy Chinese adults, with an average age of 40.15 ± 12.28 years, was selected. Participants' Fitzpatrick skin types and classifications (neutral, dry, oily, combination dry, or combination oily) were meticulously documented. All participants provided informed consent prior to study commencement. The 14‐day clinical research was carried out in a testing center, Shanghai, China. The research protocol (SHCPCH191009276) was examined and approved by the ethics committee for clinical research. Benefits, risks, and potential complications were explained to the subjects, and informed written consent was obtained from participants.

#### 2.7.2 Application Protocol

Participants were instructed to apply the placebo or test formulation twice daily after a gentle cleansing routine. Participants were randomly assigned to apply the test formulation to either the left or right side of the face, with the contralateral side applying the placebo. The placebo was devoid of active ingredients, while the test formulation incorporated PUR9‐2 (0.1% PURP plus 0.002% EQ9). The 14‐day study period mirrored typical skincare routines, with participants abstaining from the use of other efficacy‐based products to avoid confounding effects.

#### 2.7.3 Assessment Time Points

Participants underwent evaluations at baseline (Day 0), Day 7, and Day 14. Prior to each assessment, the participants' faces were cleansed and dried, followed by a 30‐min acclimatization period in a controlled environment (21°C ± 2°C, 50% ± 10%RH). Expert assessments, facial imaging with VISIA 7 and IPP, transepidermal water loss (Tewameter Hex), glossiness (VISIA 7, Glossymeter GL200, Translucency Meter), and wrinkles (VISIA 7 and IPP) were conducted.

#### 2.7.4 Expert Evaluation

Dermatological experts rated skin glossiness and dullness at baseline (Day 0), Day 7, and Day 14, based on a scale from 0 to 9 with lower scores indicating improved skin condition. Expert evaluation used a nine‐point scale: 0 = best, 1–3 = better, 4–5 = normal, 6–8 = worse, 9 = worst.

### 2.8 Statistical Analysis

For RT‐qPCR, GraphPad Prism was used for plotting. For Combination Index, CompuSyn software was used for analyzing and plotting. For clinical trials, data were analyzed using SPSS 28.0, with normality testing followed by parametric (*t*‐test) or nonparametric (rank‐sum test) statistical methods, depending on data distribution. The threshold for statistical significance was set at *p* < 0.05.

## 3 Results

### 3.1 RNA Extraction and Transcriptomic Analysis

**FIGURE 1:**
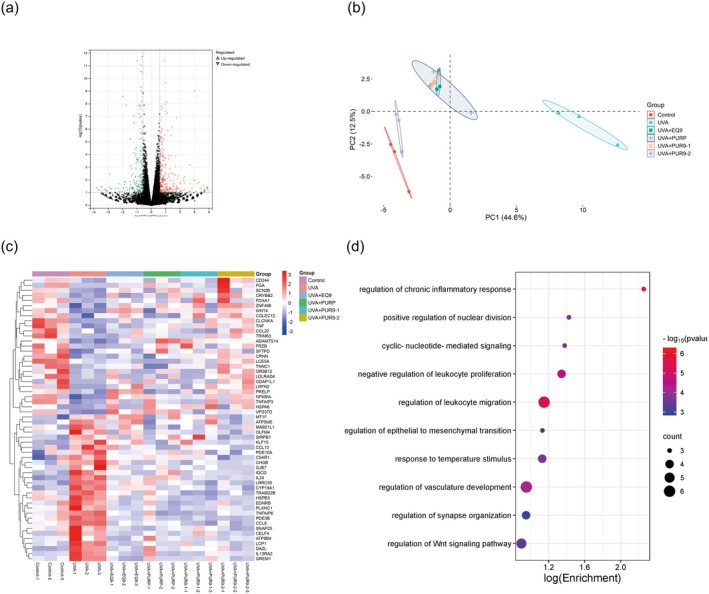
PUR9‐2 highlighted anti‐inflammatory and cell proliferation regulatory regulating genes distinct from PURP and EQ9 when used solely. (a) Differential gene expression in the UVA‐irradiated group. (b) Principal component analysis (PCA) of gene expression levels in the different groups. (c) Heatmap of gene expression levels in the different groups. (d) Functional enrichment analysis of the differentially expressed genes in the PUR9‐2 group compared to the UVA‐irradiated group.

**FIGURE 2:**
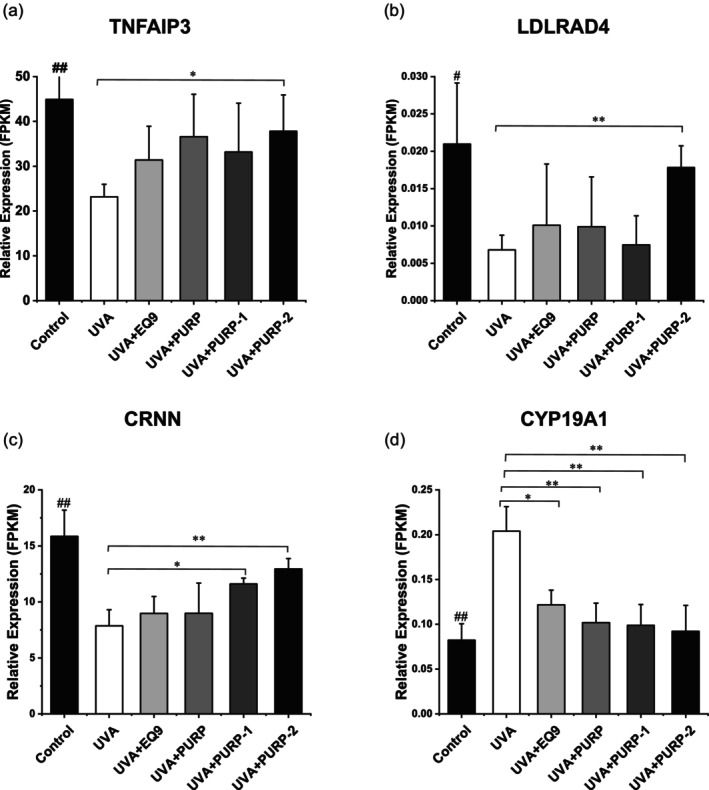
PUR9‐2 significantly increased the expression of genes relating to anti‐inflammation and barrier homeostasis. (a–d) The mean value of relative gene expression on LDLRAD4, TNFAIP3, CRNN, and CYP19A1 in control, UVA, EQ9, PURP, PUR9‐1, and PUR9‐2. The results were expressed as mean ± SD. *p < 0.05, **p < 0.01 vs. UVA group. #*p* < 0.05, ##*p* < 0.01 vs. UVA group.

First of all, by scrutinizing the transcriptomic profiling data, differential gene expression analysis yielded 467 significantly regulated genes in UVA group compared to control group, with the visualization results shown in Figure 1a (*p* < 0.1, fold change > 1.5). The gene expression‐based PCA [20] analysis revealed distinct clusters of samples corresponding to each group (Figure 1b). In particular, the cluster of samples treated with PUR9‐2 exhibited greater separation from the cluster of UVA‐irradiated samples and closer to cluster of control, suggesting a unique regulatory profile. Functional enrichment analysis based on Gene Ontology (GO) indicated that PUR9‐2 specifically targets anti‐inflammatory regulating gene sets (Figure 1c,d), setting it apart from the individual effects of PURP and EQ9. Gene expression levels by the FPKM revealed that PUR9‐2 significantly upregulated the relative gene expression of TNFAIP3 and LDLRAD4 on the UVA‐irradiated skin models, whereas EQ9 and PURP had no effects (Figure 2a,b). The combination also improved the expression of CRNN and CYP19A1 (Figure 2c,d). The result of functional enrichment analysis [13] revealed that TNFAIP3 and CYP19A1 were significantly enriched in “regulation of chronic inflammatory response” (GO:0002676, *p* = 4.99e‐07). CRNN and LDLRAD4 were enriched in “response to temperature stimulus” (GO:0009266, *p* = 0.000228) and “regulation of epithelial to mesenchymal transition,” respectively (Table 1). According to the standard GO annotations from the Gene Ontology Consortium (Table 2), TNFAIP3 (A20) was annotated to “negative regulation of NF‐*κ*B transcription factor activity” (GO:0043123, IDA) [21, 22, 23] and CRNN was involved in “positive regulation of keratinocyte proliferation” (GO:0010838, IMP) [25]. The identification of gene name in the heatmap is listed in Table S1. These preliminary findings suggest that PUR9‐2 may offer distinct skincare benefits, meriting further exploration.

**TABLE 1:** Gene function set of TNFAIP3, LDLRAD4, CRNN, and CYP19A1 based on functional enrichment analysis.

Gene	GO ID	Description	*p*
TNFAIP3	GO:0002676	Regulation of chronic inflammatory response	4.99e‐07
	GO:0070664	Negative regulation of leukocyte proliferation	3.33e‐05
	GO:0030111	Regulation of Wnt signaling pathway	0.000425
CYP19A1	GO:0002676	Regulation of chronic inflammatory response	4.99e‐07
	GO:0002685	Regulation of leukocyte migration	4.25E‐06
CRNN	GO:0009266	Response to temperature stimulus	0.000228
LDLRAD4	GO:0010717	Regulation of epithelial to mesenchymal transition	0.001443

**TABLE 2:** Gene Ontology annotations of TNFAIP3, LDLRAD4, CRNN, and CYP19A1 retrieved from the Gene Ontology Consortium.

Gene	GO ID	Category	Description	Evidence code
TNFAIP3	GO:0043124	BP	Negative regulation of canonical NF‐κB signal transduction	IDA [21, 22, 23]
	GO:2000352	BP	Negative regulation of endothelial cell apoptotic process	IDA [24]
CRNN	GO:0010838	BP	Positive regulation of keratinocyte proliferation	IMP [25]
	GO:0098609	BP	Cell–cell adhesion	IDA [26]
LDLRAD4	GO:0060392	BP	Negative regulation of SMAD protein signal transduction	IDA [27]
CYP19A1	GO:0004497	BP	Positive regulation of estradiol secretion	IDA [28, 29]

### 3.2 Quantitative RT–PCR

**FIGURE 3:**
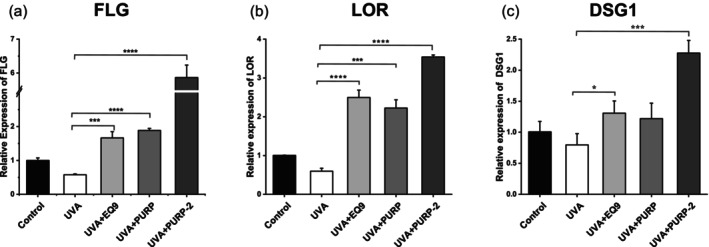
PUR9‐2 significantly increased the expression of genes relating to epidermal junction. (a–c)The mean value of relative gene expression on FLG, LOR, and DSG1 in control, UVA, EQ9, PURP, and PUR9‐2. The results were expressed as mean ± SD. *p < 0.05, ***p < 0.001, ****p < 0.0001 vs. UVA group.

RT‐qPCR [30] was then performed to assess the effect of PUR9‐2 on the expression of epidermal junctional proteins. The relative gene expression levels of FLG, LOR, and DSG1, which were decreased by UVA irradiation, were all significantly increased by EQ9, PURP, and PUR9‐2 (Figure 3). However, PUR9‐2 showed better efficacy than EQ9 or PURP. This suggests a synergistic potential for PUR9‐2 in terms of repairing the skin barrier.

### 3.3 Combination Index (CI) Analysis

**FIGURE 4:**
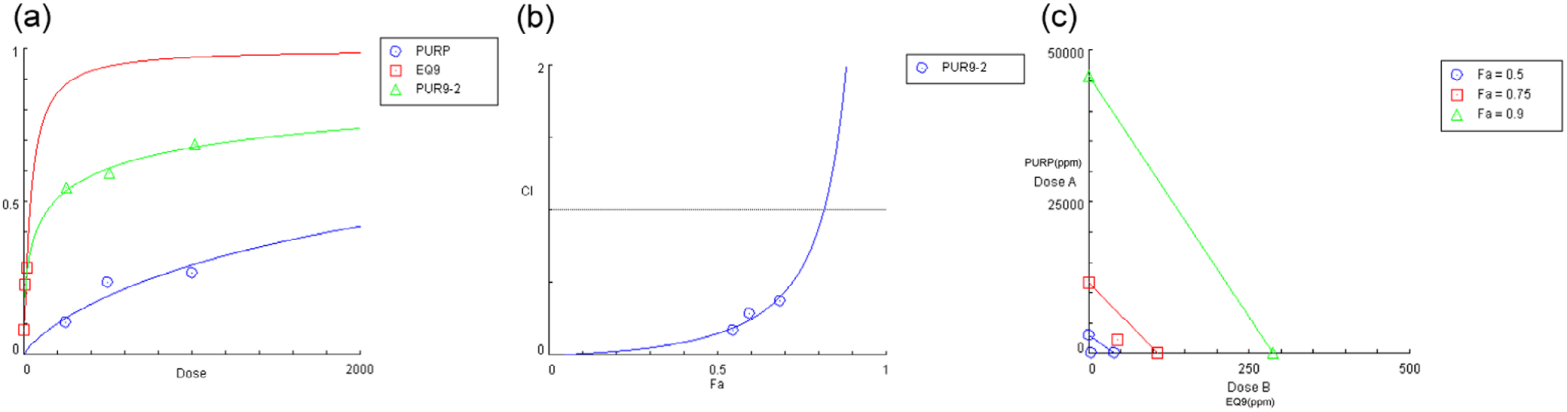
PUR9‐2 showed a synergistic effect in cell migration (CI < 1). (a) Dose–effect curve of PURP, EQ9, and Combo. (b) Combination index plot of PURP and EQ9. (c) Isobologram for 50%, 75%, and 90% effects of Combo.

The percentages of cell‐covered areas in the scratch experiment were utilized to obtain a dose–effect plot (Table S2 and Figure 4a). The median‐effect doses (Dm) of PURP, EQ9, and their combinations were determined as 2982.930 ppm, 39.579 ppm, and 180.566 ppm respectively, derived from the dose–effect plot. The synergistic effect between PURP and EQ9 was quantified using combination index (CI) and isobologram calculated by CompuSyn software based on the Chou‐Talalay method. We observed an increasing trend in the CI plot (Figure 4b), with a synergistic zone at CI < 1, and an antagonistic zone at CI > 1 when the component reached a high dosage. Isobolographic analysis revealed synergistic effects of the combination at Fa = 0.5 (CI = 0.14880) and Fa = 0.75 (CI = 0.60646). Despite the dose‐dependent synergism between PURP and EQ9 in repairing, PUR9‐2 (1000 ppm PURP +20 ppm EQ9) was proved in the synergistic zone with CI = 0.37433 (Figure 4b,c and Table S2).

### 3.4 Placebo‐Controlled Split‐Face Clinical Research

The placebo‐controlled split‐face clinical study, which enrolled 33 Chinese subjects with sensitive skin, demonstrated the change in skin parameters and subjective/objective skin radiance perception after 7 days and 14 days of topical application of a lotion containing 0.1% PURP and 0.002% nonapeptide, equivalent to the same PUR9‐2 composition and dosage as in the UVA‐induced 3D skin model evaluation.

**FIGURE 5:**
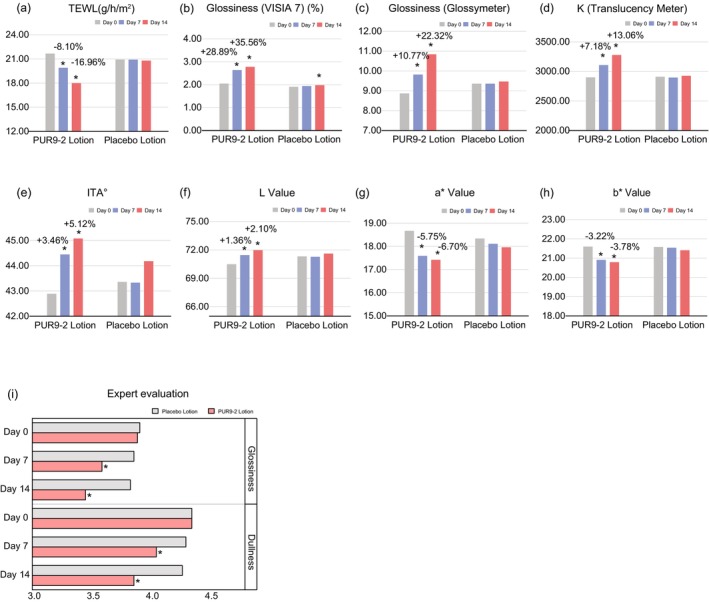
PUR9‐2 containing lotion improved translucency and glossiness of sensitive skin in a placebo‐controlled and split‐face clinical study. (a) The mean value of TEWL. (b) The mean value of *K*. (c) The mean value of glossiness detected by Glossymeter. (d) The mean value of glossiness detected by VISIA 7. Improvement (%) of Day 7/Day 14 from baseline is also presented. (e–h) The mean value of ITA°, *L*, *a*, *b*. (i) Expert evaluation of glossiness and dullness on subjects' facial skin by the PUR9‐2 lotion group and the placebo lotion group on Day 0 (baseline), Day 7, and Day 14. (Expert evaluation used a nine‐point scale: 0 = best, 1–3 = better, 4–5 = normal, 6–8 = worse, 9 = worst. *p < 0.05 vs. baseline).

Tewameter showed a significant decrease in transepidermal water loss (TEWL) of 8.10% from baseline on the half‐face treated with PUR9‐2 lotion after 7 days of twice‐daily application. This decrease tendency persisted and doubled, reaching a 16.96% reduction from baseline (Figure 5a). In stark contrast, the other half of the face treated with placebo lotion showed no significant changes in TEWL over the same period.

In addition, the Glossymeter recorded an average increase in skin glossiness on Day 7 from baseline; the VISIA 7 test confirmed these findings, showing a 28.89% increase in skin glossiness on Day 7 and an impressive 35.56% increase on Day 14 (Figure 5b,c). The translucency meter recorded a significant increase in the *K* value at Day 7, which continued to increase at Day 14. This result indicated an enhancement in translucency, which is also an indicator of skin radiance (Figure 5d). Meanwhile, both a* and b*, representing skin redness and yellowing, decreased; both L* and ITA, representing skin lightness, increased (Figure 5e–h). These effects persisted from Day 7 to Day 14 on the PUR9‐2 treated side, while the placebo treated side did not show significant changes over the same period. These results, derived from comprehensive assessments by three devices, collectively indicated that PUR9‐2 enhanced skin radiance [31].

Expert evaluation results showed that after using PUR9‐2 for 14 days, the glossiness and dullness of the subjects' facial skin had significantly improved compared to Day 0 (Figure 5i). After using PUR9‐2 for 7 and 14 days, the scores rated by experts for skin dullness were 4.05 and 3.86, and for skin glossiness were 3.59 and 3.45, respectively, showing a significant decrease compared to Day 0. There were no significant changes in skin dullness and glossiness in the placebo group from Day 0 to Day 14. Expert evaluation was based on a nine‐point scale, and the decrease in scores indicates an improvement in skin condition.

**FIGURE 6:**
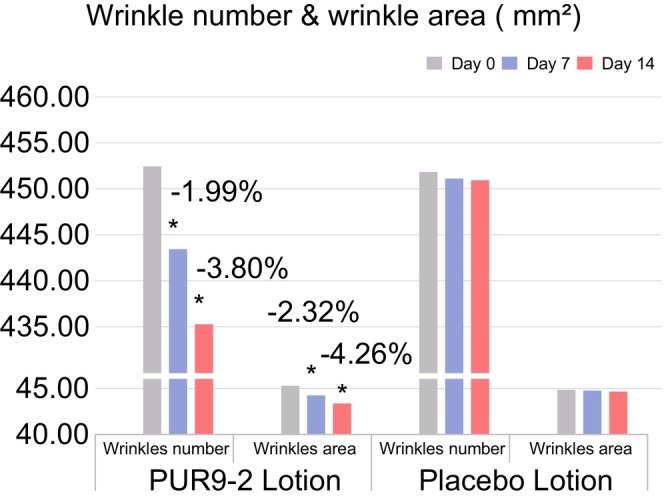
A lotion containing PUR9‐2 decreases appearance of wrinkls. The mean value of wrinkle number and wrinkle area by PUR9 lotion group and placebo lotion group on Day 0 (baseline), Day 7, and Day 14. Improvement (%) of Day 7/Day 14 from baseline is also presented (*p < 0.05 vs. baseline).

The number and area of wrinkles in the subjects were analyzed using VISIA 7 & IPP software on Days 0, 7, and 14 (Figure 6). The results showed that on the half‐face treated with PUR9‐2, both the number and area of wrinkles significantly decreased. In the PUR9‐2 group, the number of wrinkles decreased by 1.99% from Day 0 to the 7th day, and by 3.80% on the 14th day. Similarly, the area of wrinkles showed a downward trend, with reduction rates of 2.32% and 4.26% on Day 7 and Day 14. The Placebo group showed no significant changes in the number and area of wrinkles. This indicates that 14 days of PUR9‐2 use can reduce facial wrinkles.

## 4 Discussion

Our experimental data reveal distinct functional characteristics of PUR9‐2 in cutaneous modulation. PUR9‐2 demonstrated a marked capacity for skin barrier restoration on skin models and sensitive skin (Figures 3 and 5a). This phenomenon may be mechanistically linked to upregulation of CRNN gene expression (Figure 2c, Table 2). As a key regulator of epithelial differentiation and barrier homeostasis, CRNN is known to orchestrate epidermal stratification, reinforce barrier integrity, and temper inflammatory cascades [32, 33]. However, our findings also indicated that PUR9‐2 exhibited limited efficacy in suppressing melanogenesis and antioxidative activity in vitro (Figures S1 and S2). Thus, we propose that PUR9‐2 may indirectly mediate skin radiance by targeting CRNN‐associated pathways to ameliorate barrier dysfunction, rather than through conventional antioxidative or antimelanogenic mechanisms.

The decrease in the a* value treated with PUR9‐2 lotion suggests potential anti‐inflammatory properties (Figure 5g). This finding aligns with increased TNFAIP3 gene expression in vitro (Figure 2a, Table 2). As a critical regulator in cutaneous biology, TNFAIP3 negatively regulates inflammatory responses, prevents excessive inflammatory damage, apoptosis, and indirectly affects skin barrier function through inhibition of the NF‐κB signaling pathway [22, 34, 35, 36]. Thus, we hypothesize that PUR9‐2 may exert anti‐inflammatory effects through a TNFAIP3‐mediated regulatory circuit. In addition, PUR9‐2 lotion's therapeutic potential also extends to antiaging effects (Figure 6), achieving 3.80% and 4.26% reductions in wrinkle number and area respectively by Day 14 of treatment. These mechanisms may synergistically contribute to the observed enhancement of skin radiance and overall condition improvement. However, while our findings provide promising insights into the multifaceted effects of PUR9‐2 on skin health, further in‐depth studies are warranted to validate these findings.

## 5 Conclusions

In summary, the novel composition, PUR9‐2, has demonstrated potential in enhancing compromised skin radiance in a mild way and assisting in skin therapy. Transcriptomic analysis has demonstrated that the combination shows distinctive effects and is associated with regulating gene expression of the skin barrier and inflammation. It then exerted synergistic effects on keratinocyte scratch repairing and enhanced tight junction protein genes in UVA‐irradiated skin models. In the clinical study, the reduction of TEWL by PUR9‐2 lotion validated its restorative effect in compromised skin. Meanwhile, PUR9‐2 enhanced both glossiness on the skin surface, translucency within the skin structure, and skin color, showing a unique potential for improving the overall condition of compromised skin.

## Author Contributions

C.Q.: writing – original draft, formal analysis, visualization. L.Z.: writing – review and editing, conceptualization, project administration. R.Z.: writing – original draft, resources. B.W.: data curation, methodology. Q.Z.: methodology. Y.B.: resources. W.L.: data curation. L.H.: supervision. F.W.: supervision, funding acquisition. All authors have read and approved the final manuscript.

## Conflicts of Interest

The authors declare no conflicts of interest.

## Supporting information


**Figure S1.** The effects of PUR9‐1 and PUR9‐2 on melanogenesis.
**Figure S2.** The antioxidant effects of PURP, EQ9 and PUR9‐2.
**Table S1.** The identification of gene name in the heatmap.
**Table S2.** In vitro scratch wound healing and CI values of PURP, EQ9, and Combo.

## Data Availability

The data that support the findings of this study are available from the corresponding author upon reasonable request.
