# Supplementary material for: Synergistic Enhancement of Compromised Skin Radiance: A Clinical Investigation of Prinsepia utilis Royle Polysaccharides and Nonapeptide Co‐Application
Source: J Cosmet Dermatol. 2025 May 14;24(5):e70204. doi: 10.1111/jocd.70204 (PMC12077750; doi:10.1111/jocd.70204)
Supplement: Supplementary file 1 — Figure S1. The effects of PUR9‐1 and PUR9‐2 on melanogenesis. Figure S2. The antioxidant effects of PURP, EQ9 and PUR9‐2. Table S1. The identification of gene name in the heatmap. Table S2. In vitro scratch wound healing and CI values of PURP, EQ9, and Combo. [file JOCD-24-e70204-s001.docx]

**Figure S1.** **The effects of PUR9-1 and PUR9-2 on melanogenesis.**

The results were expressed as mean ± SD. ***P<0.01 vs control group.


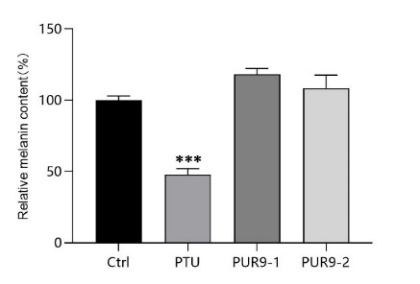


**Figure S2. The antioxidant effects of** **PURP, EQ9 and PUR9-2.**

A lower relative ratio of superoxide anion is associated with enhanced antioxidant efficacy. The results were expressed as mean ± SD. ****P<0.0001 vs control group.


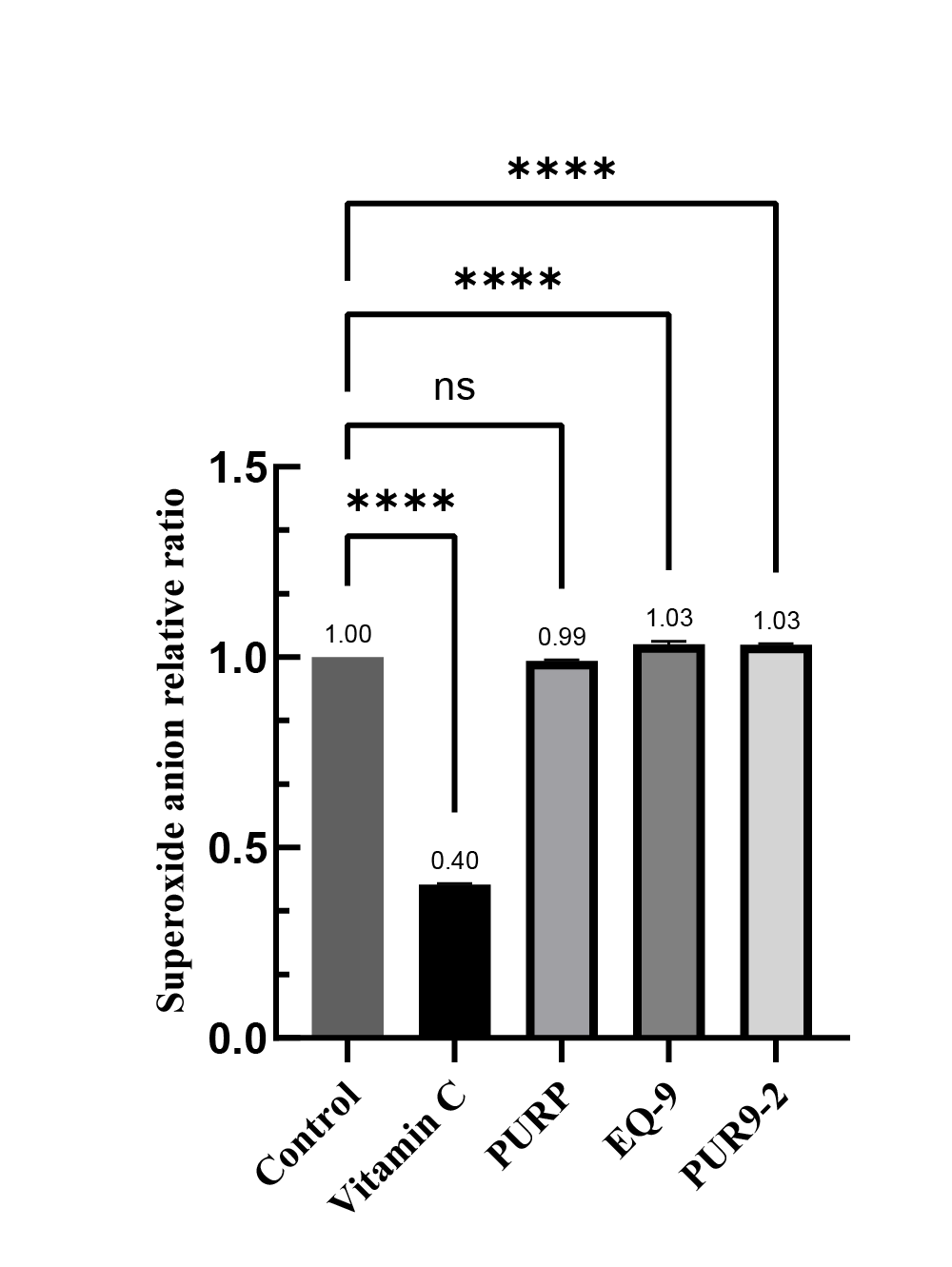


**Table S1. The identification of gene name in the heatmap.**

| Gene_ID | Name |  | Gene_ID | Name |
| --- | --- | --- | --- | --- |
| ENSG00000172362 | OR5B12 |  | ENSG00000123610 | TNFAIP6 |
| ENSG00000158022 | TRIM63 |  | ENSG00000169020 | ATP5ME |
| ENSG00000143536 | CRNN |  | ENSG00000101307 | SIRPB1 |
| ENSG00000244752 | CRYBB2 |  | ENSG00000137869 | CYP19A1 |
| ENSG00000118503 | TNFAIP3 |  | ENSG00000197405 | C5AR1 |
| ENSG00000122223 | CD244 |  | ENSG00000166923 | GREM1 |
| ENSG00000158270 | COLEC12 |  | ENSG00000164411 | GJB7 |
| ENSG00000162552 | WNT4 |  | ENSG00000162892 | IL24 |
| ENSG00000265763 | ZNF488 |  | ENSG00000152270 | PDE3B |
| ENSG00000100906 | NFKBIA |  | ENSG00000181374 | CCL13 |
| ENSG00000162998 | FRZB |  | ENSG00000166578 | IQCD |
| ENSG00000133661 | SFTPD |  | ENSG00000136040 | PLXNC1 |
| ENSG00000149575 | SCN2B |  | ENSG00000136160 | EDNRB |
| ENSG00000171560 | FGA |  | ENSG00000089199 | CHGB |
| ENSG00000188783 | PRELP |  | ENSG00000163884 | KLF15 |
| ENSG00000102962 | CCL22 |  | ENSG00000198417 | MT1F |
| ENSG00000186207 | LCE5A |  | ENSG00000269113 | TRABD2B |
| ENSG00000114854 | TNNC1 |  | ENSG00000101489 | CELF4 |
| ENSG00000176428 | VPS37D |  | ENSG00000183908 | LRRC55 |
| ENSG00000232810 | TNF |  | ENSG00000112541 | PDE10A |
| ENSG00000124194 | GDAP1L1 |  | ENSG00000108700 | CCL8 |
| ENSG00000186510 | CLCNKA |  | ENSG00000123496 | IL13RA2 |
| ENSG00000138316 | ADAMTS14 |  | ENSG00000136167 | LCP1 |
| ENSG00000168675 | LDLRAD4 |  | ENSG00000092345 | DAZL |
| ENSG00000173110 | HSPA6 |  | ENSG00000180660 | MAB21L1 |
| ENSG00000129514 | FOXA1 |  | ENSG00000104043 | ATP8B4 |
| ENSG00000156564 | LRFN2 |  | ENSG00000169271 | HSPB3 |
| ENSG00000102837 | OLFM4 |  | ENSG00000132639 | SNAP25 |

**Table S2****. In vitro scratch wound healing and CI values of PURP, EQ9 and Combo.**

| Sample Name | Total Dose | Cell-covered area±SD (%) | CI Value |
| --- | --- | --- | --- |
| Control | - | 19.974±5.531 |  |
| EFG | 1000 IU/ml | 99.706±0.469 |  |
| PURP | 1000 ppm | 26.686±14.058 | - |
|  | 500 ppm | 23.557±13.794 | - |
|  | 250 ppm | 10.651±6.345 | - |
| EQ9 | 20 ppm | 28.588±5.707 | - |
|  | 10 ppm | 23.093±3.574 | - |
|  | 5 ppm | 7.944±2.569 | - |
| Combo | 1000 ppm PURP+20 ppm EQ9 | 68.720±9.055 | 0.37433 |
|  | 500 ppm PURP+10 ppm EQ9 | 59.404±6.337 | 0.28360 |
|  | 250 ppm PURP+5 ppm EQ9 | 54.612±3.761 | 0.17349 |
